# West Nile Virus: Seroprevalence and Epidemiological Study in Blood Donors in North Central Anatolia Amid Global Threats

**DOI:** 10.1002/jmv.70900

**Published:** 2026-04-01

**Authors:** Ayse Semra Gureser, Djursun Karasartova, Nezahat Kosar‐Askin, Aysegul Taylan‐Ozkan

**Affiliations:** ^1^ Dr. Abdurrahman Yurtaslan Ankara Oncology Hospital Medical Microbiology Ankara Türkiye; ^2^ Department of Medical Microbiology Faculty of Medicine, Hitit University Corum Türkiye; ^3^ Department of Medical Microbiology, Faculty of Medicine Cyprus International University Nicosia Cyprus

**Keywords:** blood donor, IgG, seropositivity, West Nile virus

## Abstract

West Nile virus (WNV) is a pathogen belonging to the family Flaviviridae and the genus Flavivirus. This study examined WNV IgG antibodies in healthy blood donors at Hitit University Blood Centre in Corum, Türkiye, during 2019–2020 using ELISA, thereby providing seroepidemiological data on WNV in northern central Anatolia. It also investigated the associations between ABO (antigens A and B) and Rhesus (D antigen) blood group system and WNV seropositivity. Blood samples from 500 individuals (155 women, 345 men) aged 18 years or older who visited the Blood Transfusion Centre were tested using the Anti‐West Nile Virus ELISA kit (IgG) (EUROIMMUN Medizinische Labordiagnostika AG, Lübeck, Germany). Donor epidemiological and demographic info was recorded via the hospital system. WNV IgG antibody was detected in seven (1.4%) donors, comprising five females and two males, while one (0.2%) male donor had a borderline test result. Of the 500 donors, 428 (85.6%) were residents of Corum province (40°32′56″ N‐34°57′12″ E), while 72 (14.4%) came from other regions. Within the Corum group, 4 donors (0.8%) were seropositive, with no borderline results. When comparing individuals by gender, the positivity rate was 3.4% in females and 0.6% in males, a difference that was statistically significant (*p* = 0.032). WNV seropositivity was highest (5 out of 7) in the 26–45 age group, although this was not statistically significant (*p* = 1.000). The highest WNV seroprevalence was observed in donors with the O Rh(D) blood group, but this difference was not statistically significant (*p* = 0.292). WNV is circulating in our region. Additionally, women appear to be at higher risk for WNV. Our study is the first research on this subject in our area and has made a significant contribution to the literature. Clinicians in North Central Anatolia should consider this disease when assessing potential cases and adjust their diagnoses and treatments accordingly. More detailed research using advanced methods is needed to explore its seroprevalence, reservoir, and vector in the region, as well as to evaluate the implementation of routine WNV screening strategies.

## 1 Introduction

West Nile virus (WNV) was first isolated from the blood of a febrile woman in the West Nile region of Uganda in 1937 [1]. It is a mosquito‐borne pathogen belonging to the family Flaviviridae and genus Flavivirus, and recently renamed Orthoflavivirus nilense by the International Committee on Taxonomy of Viruses [2].

It primarily circulates globally via a mosquito‐bird enzootic cycle. Migratory birds play an essential role in spreading this virus into new areas. Human and equine infections usually cause encephalopathies and are transmitted by bites from infected female *Culex* mosquitoes [3]. However, due to insufficient viremia, humans and horses cannot transmit the virus and are consequently classified as definitive hosts. Additionally, transmission to humans may also occur through blood transfusion and tissue or organ transplantation [4, 5].

Approximately 80% of people infected with this virus remain asymptomatic, whereas 20% develop WNV febrile syndrome. Less than 1% progress to severe or fatal neurological manifestations, including WNV neuroinvasive disease, which may comprise acute flaccid paralysis, encephalitis, meningitis, meningoencephalitis, or a combination thereof [1].

Although fewer than 300 cases were recorded in Europe in 2017, over 1300 WNV cases and 90 deaths have been documented in 2018 [6]. The increase in cases in 2018 may be due to an unusually warm summer. Mosquitoes reproduce faster and are more active in warm weather. Higher temperatures also accelerate virus spread. On sunny days, people usually spend more time outside, which raises their risk of exposure to the vector [6]. Climate change, notably the rise in temperatures and the intensification of rainfall, has contributed to the spread of WNV throughout Europe [7, 8].

**Figure 1:**
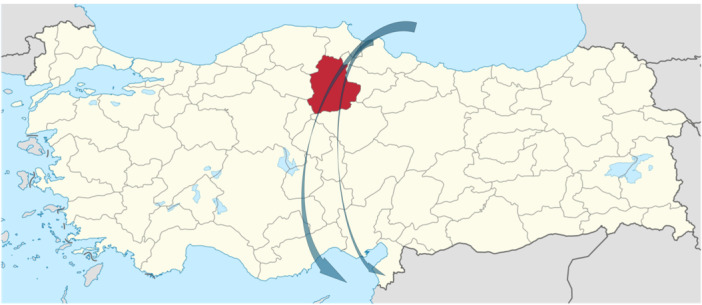
Localization of Corum (shown as red) in Türkiye. Green arrows indicate the major bird migration routes that cross Corum (This figure is adapted from Hacioglu et al. [11]).

WNV is widespread in Anatolia; however, most data on WNV circulation comes from the Aegean and Mediterranean regions, with limited information from Central Anatolia [9]. *Culex* species, the main vector of WNV, could be found across Türkiye, and Corum lies on one of the major bird migration routes, increasing the risk of WNV epidemics in this province (Figure 1) [10, 11].

Detection of specific antibodies (Immunoglobulin G [IgG]/Immunoglobulin M [IgM]) is the most common method for diagnosing human WNV infections [5, 12]. WNV IgM antibodies typically become detectable between 3 and 8 days following the onset of illness and generally remain present for a period ranging from 30 to 90 days. WNV IgG antibodies emerge shortly after IgM antibodies, approximately 4 to 16 days post‐infection, and may persist for an extended duration, potentially up to 500 days [13].

In Türkiye, routine and mandatory serological screening is conducted to prevent transfusion‐transmitted infections in blood donations. All donor samples in national blood banking practices are screened for hepatitis B virus (HBsAg), hepatitis C virus (anti‐HCV), human immunodeficiency virus (anti‐HIV 1/2), and syphilis (*Treponema pallidum*) [14]. Blood and blood products that test positive are not used clinically. Large‐scale seroprevalence studies conducted at various centers in Türkiye over the past few years show that these pathogens remain important parameters for ensuring blood safety and that routine donor screening focuses on them [15, 16]. This mandatory screening practice is widely cited in both national blood safety guidelines and various blood bank studies, and routine screening for arboviruses (e.g., WNV) is not performed outside of these parameters [14, 15, 16].

This study aimed to investigate the presence of WNV IgG antibodies in healthy blood donors presenting at the Hitit University Faculty of Medicine Blood Centre in Corum, Türkiye, during 2019 and 2020 using ELISA, and to contribute to the seroepidemiological data on WNV in the northern central Anatolia region. Furthermore, identifying the presence and extent of WNV activity in our region will raise awareness of WNV symptoms and diagnostic methods, and aid in understanding undiagnosed febrile illnesses and central nervous system infections. In the current study, the relationship between antigens A and B (ABO) and Rhesus (D antigen) [Rh (D)] blood group status and WNV seropositivity was also investigated.

## 2 Methods

The study commenced after receiving approval from the ethics committee (201935/28.08.2019, Hitit University Clinical Research Ethics Committee). Blood samples were collected at the Blood Transfusion Centre of Hitit University Medical Faculty and at Erol Olcok Training and Research Hospital. Following centrifugation, samples were stored at −80°C until analysis.

The study included 500 voluntary blood donors (155 women and 345 men) aged 18 and over who met the standard blood donation eligibility criteria defined in national legislation [14]. Individuals with acute febrile illness, known immunosuppressive conditions, incomplete demographic or clinical data, and those who tested positive for any infectious agent during routine donor screening were excluded. For analytical purposes, participants were categorized into three age groups: 18–25 years (*n* = 112), 26–45 years (*n* = 299), and 46–65 years (*n* = 89).

ABO and Rh (D) blood groups were determined using an automated Across Gel® blood grouping system based on gel‐column agglutination technology according to the manufacturer's description (DiaPro Diagnostik Urunler San. ve Tic. A.S., Istanbul, Türkiye).

Serum samples were tested using the Anti‐West Nile Virus ELISA (IgG) (Euroimmun Medizinische Labordiagnostika AG, Lübeck, Germany) according to the manufacturer's instructions. A value of < 0.8 was considered negative, ≥ 0.8 and < 1.1 were regarded as borderline, and ≥ 1.1 was considered positive.

Epidemiological and demographic information about donors was recorded in the hospital information system. Detailed information on risk factors and confirmed contact with Flavivirus species was not available.

IBM SPSS v.22 was used for statistical analysis. The mean and standard deviation of clinical parameters were calculated for all donors. The normality of the data was tested using the Kolmogorov–Smirnov and Shapiro–Wilk tests. For normally distributed data, the Student's *t*‐test was used to compare two groups; the Mann–Whitney *U*‐test was used to compare each pair of groups, and the Kruskal–Wallis test was used to compare more than two groups. For comparisons of categorical data, Pearson's chi‐square test and Fisher's exact test were utilized. Significance was assessed at *p* < 0.05.

## 3 Results

WNV IgG antibody was detected in seven (1.4%) donors, comprising five females and two males, while one (0.2%) male donor had a borderline test result.

Available donor records were retrospectively reviewed for underlying diseases, known co‐infections, and recognized risk factors. None of the WNV‐seropositive individuals had documented chronic illnesses or known co‐infections at the time of donation.

Of the 500 donors, 428 (85.6%) were residents of Corum province (40°32′56″ N‐34°57′12″ E), while 72 (14.4%) were from other regions. Within the Corum group, 424 donors (84.8%) were seronegative, 4 (0.8%) were seropositive, and no borderline results were recorded. Four residents in Corum who tested seropositive for WNV hadn't traveled outside the province in the last 5 years. WNV seropositivity was also found in three blood donors, living in Istanbul (41°0′44″ N‐28°58′34″ E), Ankara (39°55′38″ N‐32°51′52″ E), and Sanliurfa (37°9′30″ N‐38°47′30″ E) who had resided in Corum for the past 5 years but whose official residences were in other provinces, and who maintained links with these cities. Of the donors from cities other than Corum, 68 (13.6%) were negative, 3 (0.6%) were positive, and 1 (0.2%) was borderline.

When comparing individuals by gender, the positivity rate was 3.4% in females and 0.6% in males, and this difference was statistically significant (*p* = 0.032). The male‐to‐female seropositive donor ratio was 2:5. The distribution of WNV seropositivity by gender is shown in Table 1.

**Table 1:** Distribution of WNV seroprevalence by gender, among blood donors in Corum, Türkiye.

WNV IgG	Male		Female		Total	
	*N*	%	*N*	%	*N*	%
Negative	342	68.40	150	30.00	492	98.40
Positive	2	0.40	5	1.00	7	1.40
Suspicious	1	0.20			1	0.20
Total	345	69.00	155	31.00	500	100.00

*Note: p* = 0.032.

In the study group, donors' ages ranged from 18 to 56 (mean age 34.6). WNV seropositivity was highest (5/7) in the 26–45 age group, although approximately half of the donors were in this age group, and the difference was not statistically significant (*p* = 1.000). The distribution of WNV seroprevalence by age groups is summarized in Table 2.

**Table 2:** Distribution of WNV seroprevalence across age groups, among blood donors in Corum, Türkiye.

Age groups	WNV IgG						Total	
	Negative		Positive		Suspicious			
	*N*	%	*N*	%	*N*	%	*N*	%
18–25	111	22.20	1	0.20			112	22.40
26–45	294	58.80	5	1.00			299	59.80
46–65	87	17.40	1	0.20	1	0.20	89	17.80
Total	492	98.40	7	1.40	1	0.20	500	100.00

*Note: p* = 1.00.

WNV seropositivity was most frequently detected in donors with the O Rh(D) blood group. Most blood donors belonged to the A Rh (D) positive and O Rh (D) positive blood groups. The difference was not statistically significant (*p* = 0.292). The distribution of WNV seroprevalence by blood group is shown in Table 3.

**Table 3:** Distribution of WNV seroprevalence by blood group among blood donors in Corum, Türkiye.

Blood group	WNV IgG						Total	
	Negative		Positive		Suspicious			
	*N*	%	*N*	%	*N*	%	*N*	%
**O**								
**D‐**	20	4.00					20	4.00
**D**+	121	24.20	5	1.00			126	25.20
**AB**								
**D‐**	6	1.20					6	1.20
**D**+	40	8.00					40	8.00
**A**								
**D‐**	25	5.00					25	5.00
**D**+	216	43.20	1	0.20	1	0.20	218	43.60
**B**								
**D‐**	9	1.80					9	1.80
**D**+	55	11.00	1	0.20			56	11.20
**Total**	492	98.40	7	1.40	1	0.20	500	100.00

*Note: p* = 0.292.

## 4 Discussion

WNV predominantly circulates globally, with numerous factors contributing to outbreaks. These factors include international trade, travel activities, bird migration patterns, climate change, and insufficient medical preparedness [1]. The virus is widely distributed across Europe, the Middle East, and parts of Asia, and numerous seroepidemiological studies have demonstrated ongoing viral circulation in countries neighboring Türkiye [1, 5, 6, 7, 8]. Human cases were mainly reported in Southern Europe (*n* = 4546), equating to 1.54 WNV cases per 100 000 residents [1]. Greece has experienced several outbreaks since 2010, with both human cases and serological evidence of infection reported in multiple regions [17]. Similarly, WNV activity has been documented in Balkan countries, including Serbia and Romania, where both human infections and vector surveillance studies have confirmed the presence of the virus [18, 19, 20]. In Russia, annual cases of the disease have been reported since 1999, particularly in the southern European part of the country [21, 22]. In Iran, serological studies conducted among humans and animals have also indicated exposure to WNV, suggesting that the virus is established in the region [23]. One systematic review analyzing 77 studies confirms widespread WNV circulation in the Eastern Mediterranean region, with evidence in humans, animals, and vectors throughout the region [24]. Compared with neighboring countries, the 1.4% seroprevalence observed in this study appears lower than rates reported in some Balkan and Eastern Mediterranean countries, where levels ranging from approximately 3% to 10% have been documented in certain populations. Recurrent WNV outbreaks in these regions indicate sustained viral circulation. Differences in seroprevalence between countries may be related to ecological factors such as mosquito vector density, climatic conditions, migratory bird routes, and surveillance intensity.

Türkiye is situated in a region where WNV is endemic and epizootic [1, 9, 25]. In 2010, 47 human cases of WNV infection, including 12 laboratory‐confirmed and 35 probable cases, were reported in Türkiye for the first time identified through routine surveillance. The patients hailed from 15 provinces, predominantly in the western part of the country [25]. Although the initial case was documented in 2010, numerous serological investigations conducted before the human case suggest that the infection has existed in Türkiye for an extended period [9, 25, 26, 27]. Analysis of samples collected from 179 patients who applied to the outpatient clinic in the Southeastern Anatolia Region in 2005 revealed a WNV seroprevalence of 9.5% [26]. In a population‐based study conducted in Manisa in 2014, WNV IgG antibodies were detected in 3.8% of the population [27]. The Ministry of Health in Türkiye reported 107 WNV infections from 2010 to 2023 [28]. Observation of 1.4% WNV IgG seropositivity in our study appears compatible with prior evidence from Türkiye, while also supporting the possibility of regional heterogeneity. All of these findings collectively suggest that WNV exposure among healthy individuals in Türkiye remains relatively low but detectable.

Because WVV can be transmitted through blood transfusion, blood safety remains an important consideration. In several countries, particularly the United States and some European nations, routine screening of blood donors for WNV using nucleic acid testing (NAT) has been implemented to reduce the risk of transfusion‐transmitted infections [29, 30]. Following the large WNV outbreak in North America in 2002, systematic screening of blood donations significantly reduced transfusion‐associated transmission [31]. Especially, routine testing for WNV among blood donors during the transmission season is a crucial surveillance and control measure to prevent human‐to‐human transmission. In a systematic review, analysis of sera from 137 135 blood donors across three Southern European countries—Italy, Cyprus, and Türkiye—showed a pooled IgG prevalence of around 2.0% [1]. This analysis also reports a 5.6% seroprevalence across groups in Turkey, including the general population, blood donors, and hospitalized patients; however, the seroprevalence rate among blood donors was 3.2% [1]. In 2009, a study investigating WNV seroprevalence among blood donors in Central Anatolia reported a seropositivity rate of 1.8% [32]. Other studies from Ankara and Istanbul also confirm the existence of the virus in blood donors [33, 34]. In our study, WNV seropositivity was found in seven (1.4%) donors (five females and two males). In comparison, one male donor (0.2%) was considered a suspected positive. Because three of the seven seropositive individuals also resided outside Corum, the possibility that transmission occurred in these provinces cannot be excluded. Excluding these positive cases, the positivity rate in our region is estimated to be 0.8%.

In Türkiye, the available seroprevalence data suggest low‐level but measurable exposure rather than a clearly high endemic burden. For this reason, universal year‐round screening may not currently be justified solely on the basis of existing evidence; however, seasonal or risk‐based screening strategies could be evaluated in high‐risk settings after considering local epidemiology, transmission season, transfusion safety priorities, and cost‐effectiveness [29, 30, 31, 32, 33, 34].

In human studies, men are generally thought to be more likely than women to develop WNV disease [35, 36, 37, 38]. A survey of WNV infection cases from 11 tertiary hospitals in Türkiye in 2024 found that the median age was 63.3 years, with a male‐to‐female ratio of 2.6 [9]. From 2010 to 2018, a total of 3849 patients were identified in the EU and neighboring countries, with 78% confirmed as cases; the median age of WNV patients was 66 years, and the male‐to‐female ratio was 1.5 [39] Gulmez et al. reported higher seropositivity in females [27]. In our study, the male‐to‐female ratio was 2/5. Females had a 3.4% seropositivity rate, and males had a 0.6% rate, with a significant difference (*p* = 0.032). Our findings might be influenced by the higher proportion of women working outdoors, which increases their risk of mosquito bites. Further epidemiological studies are required in Türkiye to determine whether outdoor activities are linked to WNV infection.

Previous studies reported that being over 45 years old is a risk factor for seropositivity and that both seropositivity rates and overall seroprevalence increase with age [9, 27, 32, 40]. In our research, WNV IgG seropositivity was highest (5/7) in the 26–45 age group, but the difference was not statistically significant (*p* = 1.000).

Numerous studies have explored the links between blood groups and various diseases and infections, and the research into the connection between WNV disease severity and host HLA is ongoing [41, 42, 43]. It has also been suggested that WNV adheres to human red blood cells in whole blood, and that specific blood groups may be linked to the pathogenesis and outcomes of WNV disease [42, 44]. A study from Greece by Politis et al. investigated ABO, D, and Lewis blood groups, HLA alleles, and WNV Lineage 2 disease and morbidity in a cohort of 132 WNV cases between 2010 and 2013, comparing 51 339 WNV‐negative healthy blood donors and 246 healthy individuals from the same regions [42]. In this study, the authors found that A/D blood group negativity is a potential new risk factor for the development of symptoms after WNV Lineage 1 and 2 infection [42]. Another study examining the link between ABO and Rh(D) blood groups and WNV disease outcomes suggests that individuals with A and Rh(D) negative blood types might be at risk of developing symptomatic disease after WNV infection because of a genetic predisposition [45]. In our study, WNV seropositivity was most common among donors with the O Rh(D) blood group, but the difference was not statistically significant (*p* = 0.292).

This study retrospectively examined existing data on blood donors. According to national blood banking practices and Turkish Red Crescent guidelines, blood donations are not accepted from individuals with chronic diseases, infection risk factors, or conditions that are clinically contraindicated [14]. Therefore, WNV‐seropositive donors did not have a recorded chronic disease or known co‐infection at the time of donation. However, the lack of systematic recording of detailed exposure histories (e.g., mosquito contact, travel history, or occupational risks) in donor eligibility forms and during routine physical examinations is a significant limitation of the study.

## 5 Conclusion

The detection of WNV IgG antibodies among blood donors in the present study indicates that silent circulation of the virus may occur in the region. Although the seroprevalence observed in our study is relatively low, similar findings from other studies conducted in Türkiye suggest that WNV exposure among healthy populations is not uncommon. Additionally, women appear to be at higher risk for WNV. Our study is the first research on this subject in our area and has made a significant contribution to the literature, although having a retrospective design was a limitation.

Considering that WNV can be transmitted through blood transfusion, the safety of blood supplies remains an important public health concern [46, 47]. However, the implementation of routine WNV screening strategies in Türkiye should be carefully evaluated, taking into account epidemiological risk, seasonal transmission patterns, and cost‐effectiveness. Strengthening surveillance systems, conducting further seroepidemiological studies, and monitoring vector populations may provide valuable data to guide future public health strategies regarding WNV transmission and blood safety.

## Author Contributions

Aysegul Taylan‐Ozkan conceived, designed, and supervised the study. Ayse Semra Gureser, Djursun Karasartova, and Nezahat Kosar‐Askin performed the data collection and laboratory analyses. Nezahat Kosar‐Askin and Djursun Karasartova conducted statistical analysis and contributed to data interpretation. Ayse Semra Gureser contributed to data interpretation and manuscript drafting. All authors participated in manuscript writing, critically revised the manuscript for important intellectual content, and approved the final version of the manuscript.

## Conflicts of Interest

The authors declare that there is no conflict of interest regarding the publication of this paper.

## Data Availability

The data that support the findings of this study are not publicly available due to privacy and ethical restrictions, but are available from the corresponding author upon reasonable request.
